# Penalties of Public Singers

**Published:** 1874-06

**Authors:** 


### PENALTIES OF PUBLIC SINGERS.

A FACT little known to the public regarding the singing voice is, that almost all vocalists' throats are in a chronic state of inflammation, or an approach to it, which would be considered downright soreness by anybody else. A friend of mine, who is a *prima donna*, goes through life with a pair of red and swollen tonsils, which would serve me very nicely for a quinzy. Familiarity in such cases breeds contempt, as the proverb teaches, for the abnormally enlarged tonsils create no disturbance in my friend's mind. She remarks that a physician, who was unacquainted with artists' throats, would surely send her to bed if he got a peep at hers. But it is seldom necessary to caution the possessor of a singing voice to vigilance in the care of it; it is too precious a possession to be lightly guarded. A voice which, in the money sense, is equivalent to a row of brown stone fronts on Fifth avenue, or I know not how many oil wells in Pennsylvania, will be treated with the utmost respect by a wise possessor. Unfortunately, the singing voice is short lived, if it be used. The exactions of modern operas are so destructive in their effects, that it is calculated the average duration in freshness of a soprano voice is eight years, and of a tenor voice only six. The baritone is somewhat hardier, while the bass voice will generally last a lifetime; though there are well known instances of once celebrated bassos who still walk the earth in all their manly physical vigor, but sing no more.—*Olive Logan, in The Galaxy for June.*

